# Clinical characteristics and risk factors for mortality in COVID-19 inpatients in Birjand, Iran: a single-center retrospective study

**DOI:** 10.1186/s40001-021-00553-3

**Published:** 2021-07-21

**Authors:** Ghodsiyeh Azarkar, Freshteh Osmani

**Affiliations:** 1 Department of Biostatistics and Epidemiology, Faculty of Health, Birjand University of Medical Sciences, Birjand, Iran; 2 Infectious Disease Research Center, Birjand University of Medical Sciences, Birjand, Iran

**Keywords:** Survival model, Cox model, Risk factors of death, COVID-19

## Abstract

**Background:**

The coronavirus disease 2019(COVID-19) has affected mortality worldwide. The Cox proportional hazard (CPH) model is becoming more popular in time-to-event data analysis. This study aimed to evaluate the clinical characteristics in COVID-19 inpatients including (survivor and non-survivor); thus helping clinicians give the right treatment and assess prognosis and guide the treatment.

**Methods:**

This single-center study was conducted at Hospital for COVID-19 patients in Birjand. Inpatients with confirmed COVID-19 were included. Patients were classified as the discharged or survivor group and the death or non-survivor group based on their outcome (improvement or death). Clinical, epidemiological characteristics, as well as laboratory parameters, were extracted from electronic medical records. Independent sample *T *test and the Chi-square test or Fisher’s exact test were used to evaluate the association of interested variables. The CPH model was used for survival analysis in the COVID-19 death patients. Significant level was set as 0.05 in all analyses.

**Results:**

The results showed that the mortality rate was about (17.4%). So that, 62(17%) patients had died due to COVID-19, and 298 (83.6%) patients had recovered and discharged. Clinical parameters and comorbidities such as oxygen saturation, lymphocyte and platelet counts, hemoglobin levels, C-reactive protein, and liver and kidney function, were statistically significant between both studied groups. The results of the CPH model showed that comorbidities, hypertension, lymphocyte counts, platelet count, and C-reactive protein level, may increase the risk of death due to the COVID-19 as risk factors in inpatients cases.

**Conclusions:**

Patients with, lower lymphocyte counts in hemogram, platelet count and serum albumin, and high C-reactive protein level, and also patients with comorbidities may have more risk for death. So, it should be given more attention to risk management in the progression of COVID-19 disease.

## Background

Coronavirus disease (COVID-19) spread in 2019, and subsequently, severe acute respiratory syndrome coronavirus 2^1^ broke out rapidly [1]. This modern virus is principally related to pulmonary infection, hence, it can involve several organs, such as the spleen, cardiovascular, liver and stomach, and so on [2]. These patients mainly require hospitalization, intensive care unit admission, and most times need intubation [3].

In acute patients, symptoms usually develop into metabolic acidosis and multi-organ dysfunction syndrome, and it may eventually lead to death. The initial symptoms are usually dry cough, fever, and even fatigue. Also, some cases have no apparent symptoms. Recent researches declare that the most prevalent comorbidities in COVID-19 are diabetes, hypertension, and cardiovascular disease. Therefore, assessing patients’ clinical symptoms according to current influencing factors and comorbidities is important for clinicians [4, 5].

The time-to-event analysis is a series of statistical analyzing methods in which, the outcome variable is time until an event occurs. A key feature in survival analysis is that not everyone will necessarily experience the event of interest within the specified time of the study, so some individuals are censored [6, 7]. The key concept in survival analysis is the hazard function that describes the instantaneous risk of the event for an individual who has not already experienced the event. Such hazards can be included in survival models as Cox’s proportional hazards model [8, 9].

Studies of the clinical and epidemiologic characteristics of COVID-19 have been conducted in many countries. As one of the first conducted studies, Huang et al. [6] revealed clinical manifestations of COVID-19 patients and concluded that intensive care unit (ICU) patients had higher plasma levels of cytokines compared with non-ICU patients. Also, other studies compared clinical non-severe patients. As result, ICU patients were significantly older and more likely to have some comorbidities [10, 11]. Therefore, in this study, we used the Cox hazard model to provide a highly accurate risk-estimation model to guide the treatment of this disease. Moreover, the risk factors of status for COVID-19 patients were investigated among hospitalized COVID-19 patients in Birjand.

### Methods

This retrospective study was conducted at a hospital in Birjand, Southeast Iran. Systematic random sampling was done from COVID-19 inpatients from 1 March to 15 November 2020. Inclusion criteria were positive real-time reverse transcriptase-polymerase chain reaction (RT-PCR) or a clinical diagnosis made by the radiological responsible clinician based on signs, symptoms, or radiology consistent with COVID-19. Also, incomplete hospital records were excluded. A checklist was provided based on previous studies [12, 13]. These provided forms were completed by the patients’ file information. Interested variables based on prognostic indicators including a history of coronary artery disease, diabetes, and hypertension; smoking status; and blood biomarkers were extracted from electronic health records. Also, laboratory and radiographic parameters were included.

### Outcomes

The outcomes of the patients included improvement to discharge criteria or worsening to death. The patient discharge criteria and clinical type were based on COVID-19 diagnosis and treatment protocol version 7 [14].

COVID-19 death was defined as mortality for COVID-19 patients who die as a direct result of coronavirus infection but not patients who die indirectly as a result of the pandemic [15].

Hospitalization was defined as stay in a hospital for at least one day, so outpatients were not included. Due to time is in discrete days, at time zero, survival is 100%.

### Statistical analysis

Statistical analysis was done by SPSS.21 and R.3.2.2 software. First, the collected data were cleaned and the accuracy of the data was checked in partnership with the medical record office. The quantitative data were described by mean  ±  standard deviation; also, qualitative data described by frequency and percent. To compare groups, Chi-square test or Fisher’s exact test was used. To analyze whether the included variables and comorbidities were predictive of inpatients mortality, a multivariable analysis as a Cox proportional hazards model to identify risk factors associated with survival of COVID-19 patients were fitted. Also, log rank test was used for comparison of survival time by categorical variables. A *p*  <  0.05 was considered statistically significant. The packages “survival” and “cmprsk” were used in the analysis.

### Ethics approval

The requirement for written informed consent was waived in consideration of emerging infectious diseases. All procedures in this study were approved by the Ethical Committee of Birjand University of Medical Sciences, reference number: IR.BUMS.REC.1399.185.

## Results

### Demographic characteristics and laboratory parameters

From 20 May to 20 September 2020, a total of 360 patients diagnosed with COVID-19 were included after the exclusion of patients with no clinical record or missingness in the diagnosis record.

The mean age was 42  ±  3.46 years (minimum 23, maximum 97 years), minimum follow-up time was 2 days and the maximum follow-up time was 46 days and 52.4% were men. The overall mean (SD) of the patient’s survival time was 16.4 (1.3) days. The median survival time for dead patients was 8 days (IQR 5–13). 313 patients were discharged. The median length of hospital stay for those patients was 10 days (IQR 4–15; Table 1).

**Table 1:** Descriptive analysis of continuous demographic and clinical data details for patients in the hospital

Variables	Min	Q1	Median	Mean	SD	Q3	Max
LOS (days)	1.00	3.00	4.00	4.28	1.64	5.00	16
BMI, kg/m^2^	19.8	21.2	23	23.03	6.39	25.68	27.6
Age (years)	23	34.9	42.26	46.50	12.81	58.06	97
WBC (10^3^ µL)	0.60	2.80	4.10	4.96	1.82	6.10	42.80
RBC (10^6^ µL)	1.96	4.35	4.77	4.78	0.97	5.18	8.06
HGB (g/dl)	5.70	12.00	13.20	13.24	3.26	14.60	18.20
HCT (%)	18	36	40	39.61	10.88	43	61

*g/dl* grams per deciliter; *1 µL* 1 cell per microliter  =  1 cell per cubic millimeter (mm^3^)

According to the outcome, we divided the patients into two groups (the discharged group and the death group). Most of them had a low and moderate fever. A higher proportion of patients in the discharged group was referred to the hospital within 7 days of the onset of symptoms to treatment, as opposed to cases in the other group, who this period was more than 7 days from symptom onset to come to the hospital. The mean age of patients in the discharged group and death group were 68.00  ±  9.53 vs 41.36  ±  12.09), respectively. So that a significant difference was observed (*p * <  0.001).

COVID-19 cases in the death group had higher rates of upper respiratory symptoms, dyspnea, muscle pain, gastrointestinal symptoms, and comorbidities. Among clinical symptoms, significant differences were obtained in upper respiratory tract symptoms and dyspnea. Moreover, concerning sex, there were a higher proportion of males in the death group than that in the discharged group, and with a statistically significant difference. All these reports are summarized in Table 2.

**Table 2:** Comparison of clinical parameters and comorbidities in the both groups

Clinical parameters and comorbidities	Discharged patients (*n* = 298)	Death (*n* = 62)	Total (*n* = 360)	*p*
Age (years)				
< 60 years	226 (75.7%)	17 (27.1%)	199 (55.2%)	< 0.001
> 60 years	72 (24.3%)	45 (72.9%)	161 (44.8%)	
Sex				
Male	134 (45%)	37 (59.6%)	184 (51.0%)	0.023
Female	164 (55%)	25 (40.4%)	176 (49.0%)	
URS				
Yes	164 (55%)	53 (86.4%)	245 (68.2%)	0.033
No	134 (45%)	9 (13.6%)	115 (31.8%)	
Dyspnea				
Yes	113 (37.9%)	39 (62.6%)	169 (46.9%)	< 0.001
No	185 (62.1%)	23 (37.4%)	191 (51.9%)	
Headache				
Yes	28 (9.3%)	7 (11.1%)	36 (10.0%)	0.072
No	270 (90.7%)	55 (88.9%)	324 (90.0%)	
Hypertension				
Yes	39 (12.9%)	29 (46.5%)	96 (26.8%)	< 0.001
No	259 (87.1%)	33 (53.5%)	263 (73.2%)	
Diabetes				
Yes	24 (7.9%)	13 (20.2%)	47 (13.0%)	< 0.001
No	274 (92.1%)	49 (79.8%)	313 (87.0%)	
Coronary heart disease				
Yes	9 (2.9%)	11 (17.5%)	32 (8.8%)	0.014
No	289 (97.1%)	51 (82.5%)	328 (91.2%)	
Pulmonary comorbidities				
Yes	15 (5%)	7 (12.1%)	29 (7.9%)	0.32
No	283 (95%)	55 (87.9%)	331 (92.1%)	
Time to admission (days)				
> 6	130 (43.6%)	43 (68.7%)	194 (54.0%)	< 0.001
≤ 6	168 (56.4%)	19 (31.3%)	166 (46.0%)	
Hospital days (days)				
> 7	249 (83.6%)	16 (26.3%)	215 (59.8%)	< 0.001
≤ 7	48 (16.4%)	45 (73.7%)	144 (40.2%)	

*URS* upper respiratory symptoms; *p* *p* value

There were observed statistically significant differences in the majority of laboratory characteristics except for sodium and potassium between both studied groups (*p*  <  0.05). Also, death group patients had lower platelet count and lower oxygen saturation; in contrast, these patients had higher lymphocyte count and higher WBC. Liver enzymes (AST and ALT), and blood urea nitrogen was higher in the death group, illustrating that these patients have organ dysfunction to a different extent. These results are presented in Table 3.

**Table 3:** Comparison of laboratory parameters in studied groups

Laboratory parameters	*n* = 360		*p* value
	Discharged patients (*n* = 298)	Death group (*n* = 62)	
SaO_2_ (%)	93.08 ± 9.26	87.12 ± 9.09	< 0.001
Hemoglobin (g/L)	129.36 ± 19.36	122.39 ± 20.33	0.025
WBC(× 10^9^/L)	5.77 ± 2.37	9.44 ± 6.13	< 0.001
Lymphocytes (× 109/L)	1.15 ± 0.69	0.59 ± 0.29	< 0.001
Platelet count (× 10^9^/L)	198 ± 66.22	163.21 ± 84.97	0.001
C-reactive protein (mg/L)	98.79 ± 43.88	19.39 ± 17.36	< 0.001
Serum albumin (g/L)	39.47 ± 18.36	32.45 ± 4.71	0.002
ALP (U/L)	60.26 ± 19.44	82.91 ± 49.97	< 0.001
AST (U/L)	27.74 ± 17.07	55.19 ± 39.96	0.001
ALT (U/L)	26.48 ± 24.64	38.73 ± 34.04	< 0.001
Urea (mmol/L)	4.68 ± 2.06	8.93 ± 4.37	< 0.001
Creatinine (g/L)	65.84 ± 19.72	129.56 ± 29.96	0.007
LDH (U/L)	247.33 ± 87.00	486.21 ± 129.05	< 0.001
Serum sodium (mmol/L)	129.96 ± 14.25	137.88 ± 14.17	0.179
Serum potassium (mmol/L	3.71 ± 0.36	3.85 ± 1.06	0.112

*WBC* white blood cell; *AST* alanine aminotransferase; *ALB* serum albumin; *ALP* alkaline phosphatase; *ALT* alanine aminotransferase; *LDH* lactate dehydrogenase

### Survival analysis

The hospital mortality rate for COVID-19 inpatients was 17%. The mortality rate was higher among the older patients with a more comorbidities, such as diabetes and hypertension. Results of the CPH regression model showed that the hazard of mortality was significantly increased in upper respiratory symptoms, hypertension, pulmonary comorbidities, platelet count lower than 100, serum albumin lower than 35 g/L, 25-hydroxyvitamin D levels, and serum urea nitrogen more than 8 mmol/L patients. Most non-survivor patients were admitted to the ICU. So that, the hazard ratio for mortality was significantly higher in patients requiring ICU admission (*p*  <  0.0001).

**Fig. 1:**
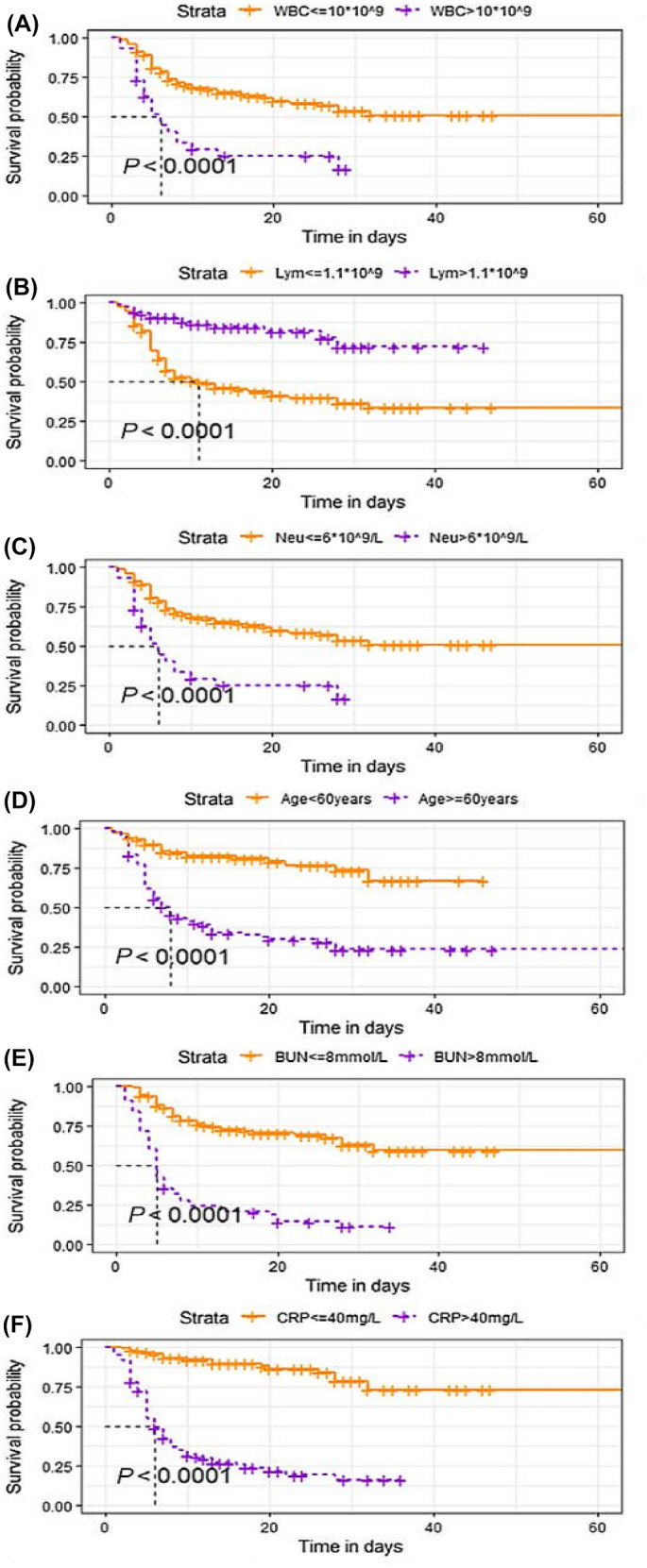
Survival curves probability based on risk factors. Survival probability in COVID-19 inpatients in different range of white blood cell (**A**), lymphocyte count (**B**), neutrophil counts (**C**), age (**D**), blood urea nitrogen (**E**) and C-reactive protein (**F**). *WBC* white blood cell; *Neu* neutrophil counts; *Lym* lymphocyte count; *AST* alanine aminotransferase; *ALB* serum albumin; *BUN* blood urea nitrogen; *CRP* C-reactive protein; *PLT* platelet count

The survival curves based on WBC count, age group, lymphocyte count, and C-reactive protein and blood urea nitrogen are shown in Fig. 1.

## Discussion

SARS-CoV-2 known as a human coronavirus is mainly transmitted by droplets and also through aerosols. The median incubation period is 4–5 days, and in the majority of them, about 12 days after infection, the symptoms gradually develop. Early in the pandemic, the period between clinical symptoms onset to progression to death is on average 14 days. Furthermore, the mortality rate was very variable at different times and area to area. This variation may be due to virus variation, besides regional differences. On the other hand, the number of patients exponentially increases. The hyperinflammatory response triggered by SARS-CoV-2 is the main reason for the death of patients [15]. Among these patients, the most usual symptoms are fever and cough, and some other patients present with sneezing; and only a few patients develop nausea or diarrhea [4]. Also, there are cases with no symptoms or tend to improve after the infection. In return, some patients appear with a fast progression, like respiratory failure or even death [14]. Also, the fatality rate significantly increased in cardiovascular patients and also patients with hypertension and diabetes [16]. Mortality increases mostly with the progression of the disease. In this study, some relevant factors and outcomes of COVID-19 inpatients were analyzed in Birjand, East of Iran to guide the treatment and prognosis of these inpatients. All included patients were classified into two categories (improved or dead) based on their disease outcome. There was a significant difference in the mean age of the two groups, indicating that older age is a risk indicator for this disease, which is often accompanied by the type of comorbidities. Previous studies showed that old age patients with comorbidities were more likely to experience a very severe phase of the disease [17]. Pathological examination showed that lesions involved multiple organs in severe patients. Some studies have reported that COVID-19 patients reveal coagulation abnormalities [18, 19]. In most cases, mild thrombocytopenia is observed in 5–41.7% of patients with COVID-19 [20]. A study showed that severe patients had a lower platelet count than non-severe cases. Moreover, the non-survivor patients had a lower platelet count than the survivors’ ones [21]. Platelets with having a substantial role in combining thrombotic and immune recruitment functions can prevent microbial invasion. Studies have shown that inflammatory markers are risk factors in COVID-19 patients [22]. Factors, such as high levels of lymphocytosis, and mononuclear macrophage infiltration, are significant markers for the rapid progression of the COVID-19 disease [23]. The current study indicated significant differences in WBC counts, and lymphocyte counts between the studied groups.

Survival analysis in this study showed that factor, such as blood urea nitrogen, was associated with a hazard of death. Also, there were significant differences in the mean value of urea nitrogen and creatinine between these groups. Another study showed that 5.1% of patients reported acute kidney injury [24]. The mechanism may be associated with the infiltration of lymphocytes in renal tissue. COVID-19 patients with renal dysfunction will have a higher risk of death, offering that efficacious renal function control should be considered in the clinical treatment. In addition, serum albumin levels somewhat reflect the body’s immune state. So a bad nutritional status can lead to lower serum albumin levels, which are detrimental to antibody production and virus clearance [7, 25]. Therefore, it should be seriously considered. Recently a study released that poor nutrition can be an effective risk factor for severe COVID-19 disease [26]. Malnutrition and anorexia probably increase the hazard of respiratory failure. This finding suggests the optimum treatment strategy in advance for older COVID-19 patients. Since the epidemic of COVID-19, much literature has reported that older age was correlated with ARDS after being infected [27]. Mortality in our study was 17·2%, which is in line with current mortality estimates for COVID-19 globally. A study recently showed a mortality rate of 26% in a large UK study [28]. In this study, we fitted the CPH model to estimate the adjusted hazard of COVID-19 inpatients. Our results showed that a higher age group has a direct effect on the hazard of death. These results are consistent with another study [29]. The findings represent that the increased mortality associated with increased C-reactive protein levels and prevalence of comorbidities (hypertension, diabetes, etc.) are also in line with other estimates, suggesting that our data are comparable with other populations [30, 31].

## Conclusion

This epidemic carries on. So, the challenges are still horrific. Evaluation of the risk factors of this disease can be beneficial for clinicians to figure out the risk of disease progression, to perform proper and efficient intervention earlier to get the best therapeutic goal, although we feel that it can be useful, in conjunction with other prognostic markers regarding clinical management decisions.

## Limitation

However carefully we prepared this study, still we had a few limitations in our study. First, our data were single-center and the sample size was not too large to demonstrate all infected people. Surely, subjective outcomes like pain may affect the assessment of our findings and lead to bias in the results. Second, patients without access to treatment were not included. Therefore, these results are only generalizable to the inpatients’ population. Also, there are missing observations in some of the recorded factors. It is possible that inaccuracies may have occurred during data collection, although our research team is experienced in collecting data.

## Data Availability

The datasets used and/or analyzed during the current study are available from the corresponding author on reasonable request.

## Abbreviations

WBC: White blood cell
Neu: Neutrophil counts
Lym: Lymphocyte count
AST: Alanine aminotransferase
ALB: Serum albumin
BUN: Blood urea nitrogen
CRP: C-reactive protein
PLT: Platelet count
CPH: Cox proportional hazard

## Footnotes

1 (SARS-CoV-2).
